# Physiological Temperance Society of the University of Louisville

**Published:** 1848-03

**Authors:** 


### PHYSIOLOGICAL TEMPERANCE SOCIETY OF THE UNIVERSITY OF LOUISVILLE.

We are happy to be able to state, that this society, after the lapse of five years, is still active and prosperous. Its meetings are held once a fortnight in the Hall, when a lecture is delivered, on the pathological effects of alcoholic drinks and other narcotic stimulants. Seven hundred and ten medical students have become members of the society. On the 14th of last month, an excellent annual discourse was delivered by Dr. Hugh Rodman, of Lagrange, in this State, one of the original members of the society. We cannot but regret, that the professors of all our schools, do not promote the establishment of similar associations; so as to familiarize the minds of their students with the whole subject of intemperance, as a cause of many diseases of the body and disorders of mind; and thus greatly increase their usefulness, while they would be themselves, secured against temptation.

